# Complimentary electrostatics dominate T-cell receptor binding to a psoriasis-associated peptide antigen presented by human leukocyte antigen C∗06:02

**DOI:** 10.1016/j.jbc.2023.104930

**Published:** 2023-06-15

**Authors:** Sushma Anand, Dene R. Littler, Jesse I. Mobbs, Asolina Braun, Daniel G. Baker, Luke Tennant, Anthony W. Purcell, Julian P. Vivian, Jamie Rossjohn

**Affiliations:** 1 Infection and Immunity Program & Department of Biochemistry and Molecular Biology, Biomedicine Discovery Institute, Monash University, Clayton, Victoria, Australia; 2 Janssen Research & Development, LLC, Philadelphia, Pennsylvania, USA; 3 Institute of Infection and Immunity, School of Medicine, Cardiff University, Cardiff, UK

**Keywords:** psoriasis, major histocompatibility complex (MHC), antigen presentation, autoimmunity, HLA-C∗06:02, T-cell receptor

## Abstract

Psoriasis is a chronic skin disease characterized by hyperproliferative epidermal lesions infiltrated by autoreactive T cells. Individuals expressing the human leukocyte antigen (HLA) C∗06:02 allele are at highest risk for developing psoriasis. An autoreactive T cell clone (termed Vα3S1/Vβ13S1) isolated from psoriatic plaques is selective for HLA-C∗06:02, presenting a peptide derived from the melanocyte-specific autoantigen ADAMTSL5 (VRSRRCLRL). Here we determine the crystal structure of this psoriatic TCR–HLA-C∗06:02 ADAMTSL5 complex with a stabilized peptide. Docking of the TCR involves an extensive complementary charge network formed between negatively charged TCR residues interleaving with exposed arginine residues from the self-peptide and the HLA-C∗06:02 α1 helix. We probed these interactions through mutagenesis and activation assays. The charged interface spans the polymorphic region of the C1/C2 HLA group. Notably the peptide-binding groove of HLA-C∗06:02 appears exquisitely suited for presenting highly charged Arg-rich epitopes recognized by this acidic psoriatic TCR. Overall, we provide a structural basis for understanding the engagement of melanocyte antigen-presenting cells by a TCR implicated in psoriasis while simultaneously expanding our knowledge of how TCRs engage HLA-C.

The chronic recurring inflammatory skin disease psoriasis affects 2 to 3% of the global population (1); the majority of cases are classified as *psoriasis vulgaris* and are associated with the formation of hyperproliferative keratinocyte plaques. Such plaques are characterized by infiltration of stimulated cutaneous T cells that become active in the absence of any identified etiological agent (2, 3, 4, 5). For those suffering the disease, formation of psoriatic lesions is acutely obvious, reducing the quality of life alongside associated comorbidities (6).

The exact mechanism(s) underlying the development of psoriasis are not fully delineated. The disease displays both autoimmune and autoinflammatory characteristics (7). Current treatments targeting TNFα or IL17/IL23 have shown to be successful in limiting the severity of symptoms (8). Environmental factors including prior streptococcal infection may also play a role in disease onset (9).

Typical of systemic autoimmune disorders, an individual’s progression toward psoriatic disease likely involves multiple factors. Nonetheless, genome-wide association studies have defined a strong genetic component to psoriasis with greater than twenty risk regions characterized. Psoriasis susceptibility locus 1 (PSORS1) has the highest risk association with susceptibility subsequently mapped to the gene for *HLA-C*. Approximately 60% of psoriasis patients express the HLA-C∗*06:02* (HLA-Cw6) allele, and these individuals often show an early disease onset (3, 10). This encodes a protein that is part of the major histocompatibility complex (MHC) class I locus. HLA-C is one of the three highly polymorphic genes termed the classical human leukocyte antigen (HLA)-I (*HLA-A*, *HLA-B*, and *HLA-C*) that are ligands for αβ T-cell receptors, killer-cell immunoglobulin-like receptors, and leukocyte immunoglobulin-like receptors.

Although genetic associations with autoimmune disorders are often compelling, identifying underlying molecular “triggers” or associated autoantigens for T cell–mediated autoimmune disorders has proved challenging (11). Insulin epitopes have been identified for diabetes (12, 13) and as potential epitopes presented by HLA-II molecules recognized by CD4+ T cells. The presentation of citrullinated self-epitopes by HLA-DRB1∗04:01/04 may contribute to rheumatoid arthritis (14, 15). Psoriasis is noteworthy as it is one of the few autoimmune disorders associated with HLA-C.

The healthy and diseased peptide repertoires presented by human HLA-A and HLA-B allomorphs have historically been preferentially studied due to their higher abundance (16, 17). In contrast, studies on HLA-C have been relatively under-represented in terms of disease association, antigen preference, activation mechanisms, and structural data. Nevertheless, HLA-C is involved in key T cell– or natural killer cell–mediated interactions including viral immunity, feto-maternal interface (18), and psoriasis (19). HLA-C presents a less diverse peptide repertoire than other HLA allomorphs and is biased toward displaying 9-mer protein fragments with hydrophobic residue anchors at P9 (20).

Searches for potential psoriatic *HLA-C∗06:02*–restricted autoantigens have yielded candidates derived from skin-related proteins such as antimicrobial LL-37 (21), the ADAMTSL5 melanocyte protein (22), and streptococcal molecular mimics of keratin (23, 24). Guided by mass spectrometry and structural approaches, we developed an *in silico* ranking system to assess HLA-C∗06:02 presentation potential (20). This helped us prioritize a previously identified psoriatic autoantigen ^67^VRSRRCLRL^75^ from ADAMTSL5 as previously identified (22).

Autoreactive CD8^+^ T cell infiltrates of psoriatic plaques have a reported preference for TRBV6-5 gene usage (25, 26). Lesional skin tissue of one HLA-C∗06:02^+^ psoriasis patient was previously used to identify the CD8^+^ T cell clone named in literature as Vα3S1/Vβ13S1 (22, 27), which utilizes a TRBV6-5∗01/TRAV17∗01 pairing. This T cell receptor (TCR) is activated by HLA-C∗06:02^+^ primary melanocytes but not HLA-C∗06:02^-^ ones or HLA-C∗06:02^+^ keratinocytes (22, 27). The highly charged epitope ^67^VRSRRCLRL^75^ from ADAMTSL5 was subsequently shown to activate this psoriatic Vα3S1/Vβ13S1 TCR (22). This finding implied a potentially increased role for melanocytes over keratinocytes as the autoimmune targets for psoriatic CD8^+^ T cells (22) but does not rule out keratinocyte targeting in other patients. We sought to further characterize this TCR recognition of HLA-C∗C6:02.

Prior studies implied that arginine residues within the melanocyte autoantigen epitope were necessary for activation of the Vα3S1/Vβ13S1 TCR (22). Of the four charged residues present, we had previously demonstrated potential TCR-facing orientations for P4, P5, and P8, while P2-Arg instead acts as a buried anchor required for HLA loading within this allotype (20). Here we present the structure of the complex between the Vα3S1/Vβ13S1 TCR bound to HLA-C∗06:02. The docking mode for this psoriatic TCR utilizes an extensive complementary charge network engaging all of the epitope’s outwards facing arginine residues alongside two others of the HLA-C α1 helix. The flexible arginine-dominated electrostatic surface of the antigen and its antigen-presenting molecule is matched by complementary charge within the TCR complementary determining region (CDR) loops. The importance of this atypical charge network was confirmed by alanine-scanning experiments and cellular activation assays. Our work suggests that psoriasis-associated TCRs directed towards HLA-C∗06:02 may need to accommodate a more charge-dominated peptide-HLA interface.

## Results

### Psoriatic pHLA:TCR cocomplex

We sought to define what features make a psoriatic TCR such as Vα3S1/Vβ13S1 (22, 27) reactive toward skin-specific autoantigens presented by HLA:C∗06:02. This TCR utilizes a TRBV6-5∗01 β-chain (TCRVβ13S1) whose nongermline-encoded CDRs are dominated by negatively charged glutamate residues paired to a glycine-rich CDR3α loop within the TRAV17∗01-encoded α-chain (TCRVα3S1). We expressed and purified the extracellular domains of this Vα3S1/Vβ13S1 TCR as well as HLA-C∗06:02 loaded with an epitope mimic from ADAMTSL5 (20). The cysteine within the native peptide sequence ^67^VRSRRCLRL^75^ was replaced with aminobutyric acid in order to facilitate HLA loading (20), an exchange that will hereafter be denoted through use of an italic bold “***C***”. This is a bioisosteric replacement that maintains the hydrophobic interaction potential of cysteine but lacks its ability to form disulfides (28).

**Figure 1:**
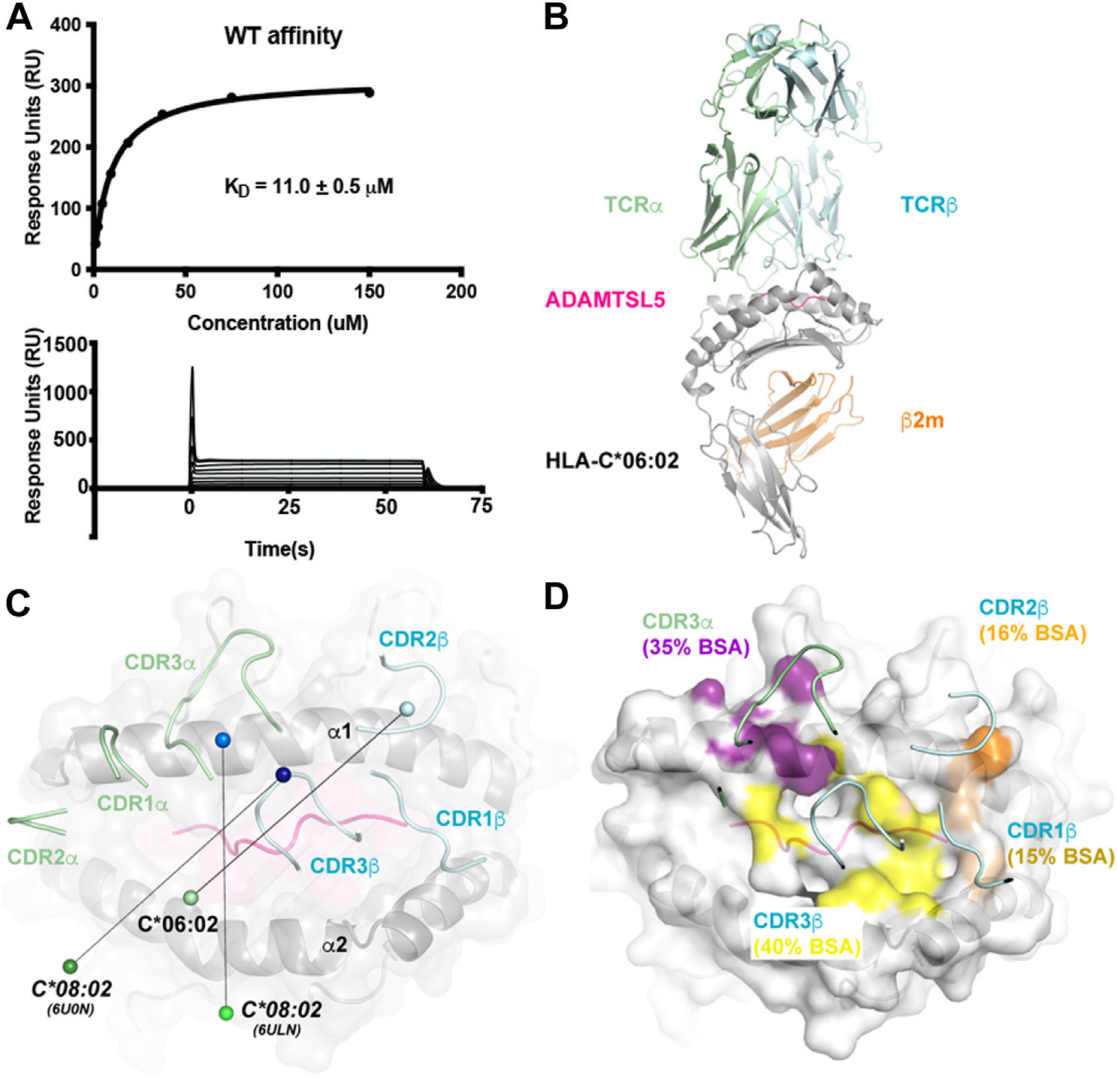
**Cocomplex of the Vα3S1/Vβ13S1 TCR HLA-C∗06:02 bound to ADAMSTL5.***A*, measurement of the binding affinity, as determined by surface plasmon resonance, between the psoriatic Vα3S1/Vβ13S1 TCR and HLA-C∗06:02 presenting the ADAMSTL5 epitope (experiment was performed in duplicate). *B*, cartoon representation of the crystallographic cocomplex structure with HLA-C∗06:02 colored *gray* and partially transparent, β2m in *orange*, and the ADAMSTL5 peptide in *pink* with the TCR α- and β-chains in *light green* and *cyan*, respectively. *C*, surface representation of the antigen presentation platform seen by the TCR, highlighting the positions of its CDR loops as well as the overall orientation of the docking mode. The center-of-mass for the TCRs’ variable domains is shown with *colored spheres*. The equivalent representation for two other published HLA-C∗08:02 TCR complexes is shown. Docking angles were calculated based on a hypothetical line drawn between the epitope’s two termini. *D*, a footprint analysis of CDR loop contributions to the interface, TCR-proximal regions of the pHLA surface are colored according to which CDR loops they contact. CDR, complementary determining region; HLA, human leukocyte antigen.

We next assessed binding of the Vα3S1/Vβ13S1 TCR to HLA-C∗06:02 loaded with VRSRR***C***LRL *via* surface plasmon resonance. HLA-C∗06:02-VRSRRCLRL was immobilized on a surface plasmon resonance chip over which increasing concentrations of recombinantly-produced TCR was passed (Fig. 1*A*). Duplicate experiments produced response curves that allowed modeling of an association with a K_D_ of ∼11 ± 0.5 μM, which falls within the range of standard TCR–peptide major histocompatibility complex interactions (29).

This affinity of this interaction was sufficient to form stable Vα3S1–Vβ13S1 TCR cocomplexes which co-eluted with HLA-C∗06:02 loaded with ^67^VRSRR***C***LRL^75^ during gel filtration. We were subsequently able to grow crystals, collect X-ray diffraction data to 2.9 Å resolution, and solve the cocomplex structure (see Fig. 1*B*; Table 1, Experimental procedures for details). The final model consisted of two copies of peptide human leukocyte antigen (pHLA)-TCR within the asymmetric unit; both have identical structure proximal to the peptide-binding groove but display a degree of divergence in the positioning of their TCR constant regions, with a final RMSD of 1.2 Å over 805 C_α_.

**Table 1:** X-ray diffraction data collection and refinement statistics

Parameter	Vα3S1/Vβ13S1:HLA-C∗06:02– VRSRR*C*LRL
Data Collection	
Space group	I222
Cell dimensions	
a, b, c (Å)	106.0, 193.0, 211.5
α, β, γ (°)	90, 90, 90
Resolution range (Å)	40–2.90 (3.06–2.90)
*R*_pim_	8.3 (70.5)
Mn(I) half-set correlation CC(1/2)	0.992 (0.44)
I/σI	8.4 (1.3)
Total observations	335,095 (49,553)
Unique reflections	48,398 (7008)
Completeness (%)	100 (100)
Multiplicity	6.9 (7.1)
Refinement	
Rwork	24.3
Rfree	29.2
Biological complexes in ASU	2
Number of atoms	
Protein	12,913
Water	0
Average B factor (Å^2^)	80.1
Bond lengths (Å)	0.012
Bond angles (°)	1.65
Ramachandran plot	
Ramachandran allowed (%)	99.5%
Ramachandran outlier (%)	0.5%

R_p.i.m_ = Σ_hkl_ [1/(N-1)]^1/2^ Σ_i_ | I_hkl, i_ - <I_hkl_> |/Σ_hkl_ <I_hkl_>.

R_factor_ = (Σ | |F_o_| - |F_c_| |)/(Σ |F_o_|) - for all data except as indicated below.

5% of data was used for the R_free_ calculation.

Values in parentheses refer to the highest resolution bin.

Binding of the TCR does not significantly alter the structure of the underlying pHLA platform (r.m.s.d. of 0.5 Å) except to the extent that some surface-exposed side chains become enveloped by the CDR loops. The psoriatic TCR-binding interface is extensive and heavily focused over the HLA-C∗06:02 α1 helix combined with interactions near the epitope midpoint (Fig. 1*C*). Total buried surface area following binding is 1830 Å^2^, with ∼66% of the contact made to the HLA platform and ∼34% to the ADAMTSL5 peptide (Fig. 1*D*). TCR docking occurs at a ∼45° angle to the HLA platform’s α1 helix with the β-chain IgV centered over it and the TCR α-chain over the α2 helix (Fig. 1*C*).

### Comparison with other HLA-C complexes

Our observed TCR docking orientation sits more centrally over HLA-C than that observed for the other characterized HLA-C–specific TCR docking modes characterized to date (Figs. 1*C* and S1) (30). The other published examples target HLA-C∗08:02 making this, as far as we are aware, the first reported example of a crystal structure of a TCR-HLA-C∗06 complex. The interfacing CDR loops of Vα3S1/Vβ13S1 are more TCR β-chain focused, leaving its TCR α-chain relatively raised such that only its CDR3α loop is mediating contacts with the HLA-C–peptide complex (Fig. 1*D*). Binding of Vα3S1/Vβ13S1 thus occurs primarily *via* the three TCR β-chain loops, while the glycine-rich CDR3α wrapped around the external face of the HLA α1 helix (Fig. 1*D*). The ranking of contributions to the interface buried surface area are CDR3β (40%) > CDR3α (28%) > CDR2β (16%) ∼CDR1β (15%). At a global level, the TCR docking angle and CDR loop contributions all fall within ranges observed previously in other HLA-A or HLA-B structures (29).

### Overview of the CDR loop contacts to HLA-C∗06:02-VRSRRCLRL

**Figure 2:**
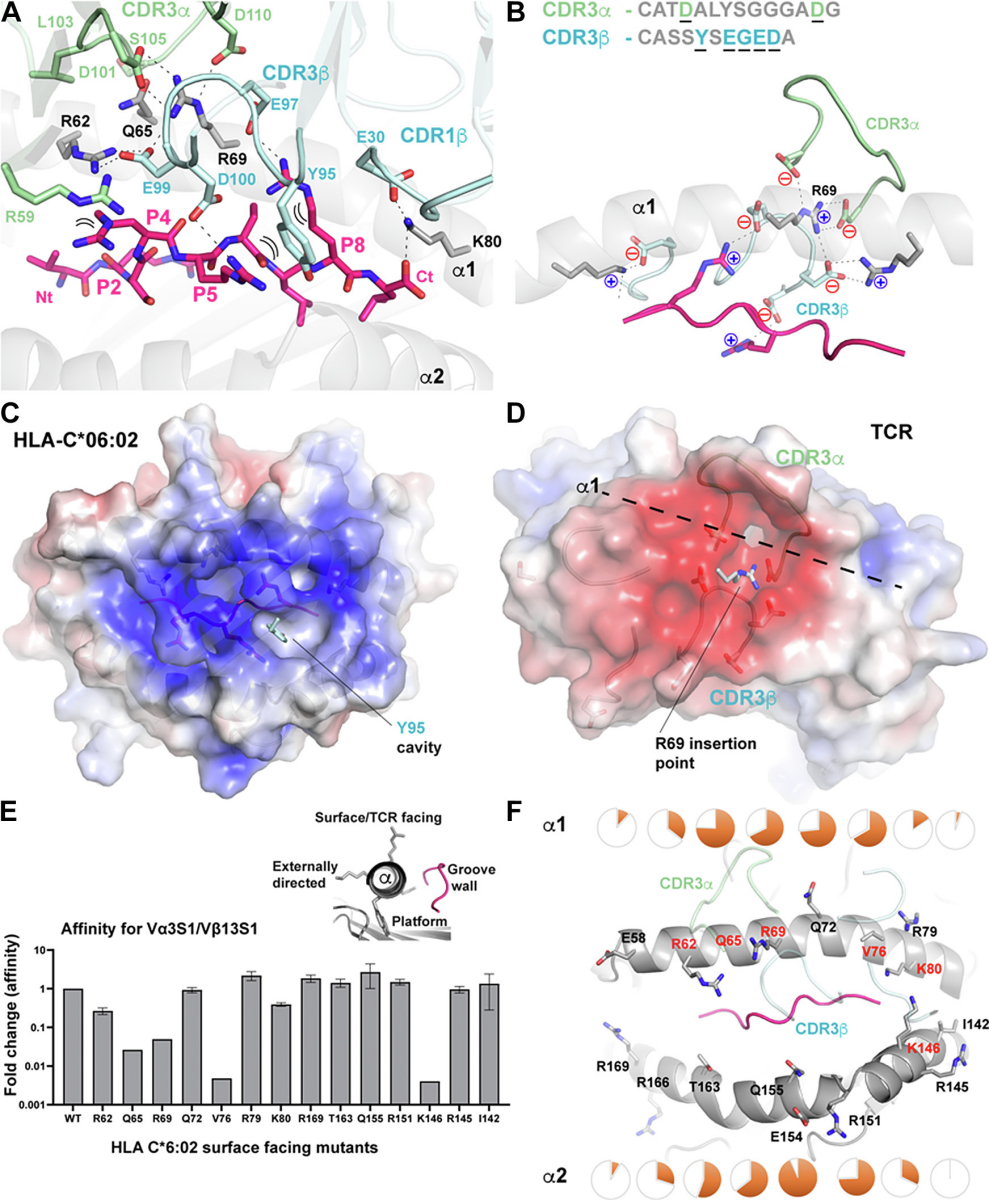
**Charge analysis and TCR alanine scanning mutagenesis.***A*, *stick* representation of the residue interactions facilitating TCR docking, *dashed lines* highlight H-bond or salt-bridge interactions. *B*, enhanced view of the Vα3S1/Vβ13S1 TCR bound to HLA-C∗06:02 near the α1-helix highlighting salt-bridges formed across the interface. Sequences of the CDR3 loops are shown; residues contacting the pHLA are *underlined*. APBS electrostatic surface potential of the (*C*) pHLA and (*D*) Vα3S1/Vβ13S1 TCR observed following separation of the interface. The underlying position for key charged residues is shown and labeled. *E*, surface plasmon resonance affinity assays displaying affinity differences for Vα3S1/Vβ13S1 binding to HLA-C∗06:02 WT and alanine mutants within the α1 and α2 helices. Ratio of measured SPR affinities relative to that determined for the WT pHLA:TCR interaction. *Upper insert:* names used to describe orientation of MHC α-helical residues. *F*, the position of TCR facing HLA-C∗06:02 α-helical residues some of which were mutated are shown and labeled. The position of interacting Vα3S1/Vβ13S1 loops is overlayed with partial transparency. Alanine mutants proven to reduce binding are labeled in *red*. Pie charts above each residue indicate the percentage of equivalent residues involved in contacts within class I pHLA:TCRs. CDR, complementary determining region; HLA, human leukocyte antigen; MHC, major histocompatibility complex.

HLA-C∗06:02 presents the ADAMTSL5 epitope such that the P4, P5, and P8 arginine residues extend upward from its presentation platform (20). Upon TCR binding, the latter two charged residues form an extensively intermingled series of salt bridges (Fig. 2, *A* and *B*). This charge-rich engagement mechanism is centered around the curled acidic CDR3β loop with the sequence CASSYSEGEDE^101^. The last germline residue from this sequence, ^TCRβ^Tyr-95, inserts its aromatic ring between the extending alkyl chains of the epitope’s P5 and P8 arginine residues. This guides these relatively mobile residues to form salt bridges to acidic CDR3β loop residues (Fig. 2*A*). Alternating charge interactions then also interface with the HLA-C∗06:02 helices with ^TCRβ^Glu-99 inserting between ^C∗06^Arg-62 and ^C∗06^Arg-69. This yields a line of four alternate charges due to a bookended interaction with CDR3α ^TCRα^Asp-101 and ^TCRα^Asp-110 (Fig. 2*B*).

HLA-C allomorphs are associated with NK cell reactivity leading to clinical genotyping into two groups: C1 allotypes carry an asparagine at residue 80, whereas the C2 group contains a lysine. Both polymorphisms contact the C-terminal carboxyl group of peptide antigens. We observe this standard interaction which the TCR registers *via* an overlaying salt bridge between ^C∗06^Lys-80 and ^TCRβ^Glu-30 (Fig. 2*A*).

On the N-terminal side of the epitope, a guanidium π-stacking interaction occurs between the epitope P4-Arg and a framework ^TCRα^Arg-59 (Fig. 2*A*). Overall, TCR docking does not require significant changes to the epitope structure. The aminobutyric acid residue substituted in place of the ADAMSTL5 cysteine was previously shown to be oriented toward the HLA (22). We observe no direct contacts to the TCR upon complexation.

In conclusion, TCR recognition of the ADAMSTL5 epitope is heavily focused on the side chains of the P4-Arg, P5-Arg, and P7-Arg.

### Electrostatic analysis of the interface

We next independently calculated the electrostatic potentials for the interfacing surfaces of the peptide HLA-C∗06:02 and the Vα3S1/Vβ13S1 TCR. The result clearly demonstrates that for this psoriatic TCR, docking is largely driven by a pHLA antigen-platform presenting a positive surface (Fig. 2*C*) that associates with a complementary series of acidic-rich CDR loops (Fig. 2*D*).

Acidic TCR residues derived from both chains’ CDR3 loops (underlined residues Fig. 2*B*) serve to make contact with positively charged peptide or α1-helical residues (Fig. 2*A*, Table S1). Another major contributor to the interface is ^TCRβ^Tyr-95 from the CDR3β loop. This aromatic key residue inserts across the interface into one of the larger neutral cavities displayed by the HLA (Fig. 2*C*), while ^C∗06^Arg-69 from HLA-C traverses the other way into a cluster of acidic TCR residues (Fig. 2, *A* and *D*). The interactions across the interface are thus predominantly charge-charge–based salt bridges.

The Coulomb attraction between two ions can be enhanced by hydrophobic shielding, reducing solvent/ion exposure. Residues within Vα3S1/Vβ13S1 provide a degree of such shielding over the α1/α2 helices and near the epitope’s C terminus. Dielectric shield residues include ^TCRα^Leu-103, ^TCRα^Tyr-104, ^TCRβ^Ala-52, ^TCRβ^Ile-54, and ^TCRβ^Tyr-95 (Table S1). The interface is more accessible at the other end of the peptide making this region more susceptible to modulation through exposure to solvent.

In conclusion, psoriatic TCR recognition of HLA-C∗06:02 involved an extensive novel network of complementary electrostatic charges.

### Alanine scanning mutagenesis

Residues deriving from the HLA-C∗06:02 α-helices contribute to the binding interface. To assess their contribution to affinity, we mutated the residues displayed on the upper TCR-facing surface of the HLA α-helices to alanine. All 14 mutants were expressed and purified similarly to WT allowing the surface plasmon resonance assays to be undertaken (Figs. 2*E* and S2, Tables S2 and S3).

Loss of either ^C∗06^Arg-62 or ^C∗06^Arg-69 completely abrogate binding (Fig. 2*E*); both central elements of the interleaving charge-network (Fig. 2*A*) completely abrogated binding (Fig. 2*E*). In contrast, an alanine mutation at the C-group polymorphic position, ^C∗06^Lys-80, retained ∼0.3-fold affinity of the WT protein (Fig. 2*E*). Its salt bridge interaction with ^TCRβ^Glu-30 is at a less-central interface position.

For the most part, measured affinities were equivalent or better than WT HLA for residues remaining distal from the TCR upon cocomplexation (Fig. 2*F*). The α2 helix is not contacted by the TCR, and ^C∗06^R151A, E154A, E166A, and R169A mutants had minimal discernible effect upon binding. Within the α1 helix the ^C∗06^R79A mutant showed no discernible effect upon binding, this resides at the C-terminus of the helix whereas Vα3S1/Vβ13S1 binds more centrally (Fig. 2*F*).

Our alanine scanning experiments confirm that residues near the midpoint of the α1 helix of HLA-C∗06:02 are important to docking of the Vα3S1/Vβ13S1 TCR.

### Activation assays

We next sought to ascertain what residues within the ADAMSTL5 epitope were required for T cell activation. Full-length Vα3S1/Vβ13S1 TCR was retrovirally transduced into the SKW3T cell line, and HLA-C∗06:02 transfected into antigen-presenting 721.221 cells. This 721.221 Cw6 cell line was pulsed with a range of self-peptides over a concentration range of 0 to 100 μg/ml. Experiments were performed with the ADAMTSL5 peptide (VRSRR***C***LRL) and surface-exposed arginine variants at P4A (VRSAR***C***LRL), P5A (VRSRA***C***LRL), or P8A (VRSRR***C***LAL) alongside C2CD, a mimotope with sequence (LRAGRSRRL), and a negative control TRAT peptide from HCMV (TRATKMQVI).

**Figure 3:**
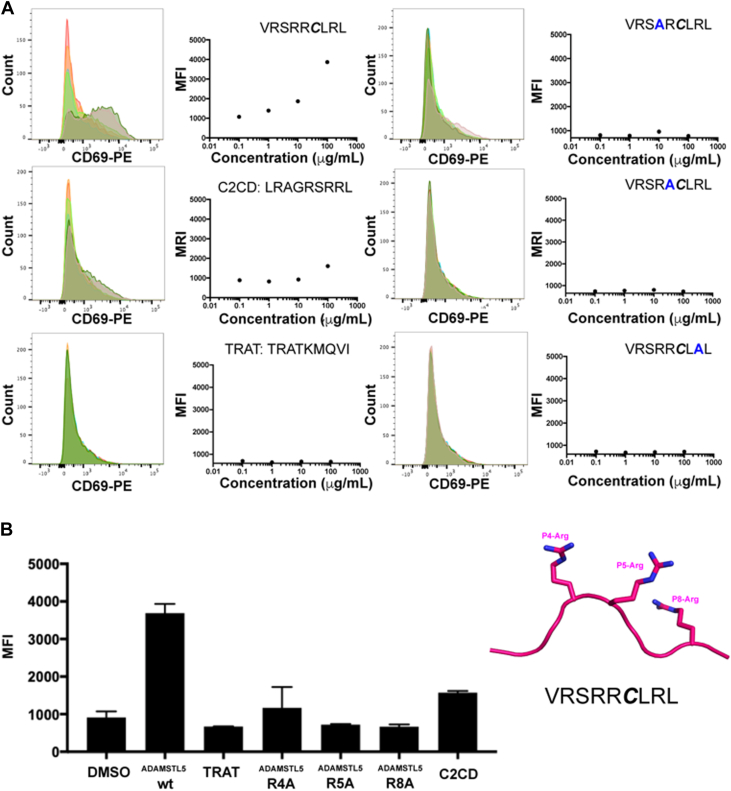
**T cell CD69 upregulation following peptide pulsing of antigen-presenting cells.***A*, on the *left* are histograms of CD69-PE–stained SKW3-Vα3S1/Vβ13S1 coincubated with 721.221_Cw6 antigen-presenting cells pulsed with the peptides indicated at increasing concentrations. On the *right* of each histogram, the mean fluorescent intensity (MFI) was recorded and plotted against peptide concentration. *B*, the CD69-PE mean fluorescent intensity recorded for SKW3-Vα3S1/Vβ13S1 incubated with 721.221 Cw6 cells pulsed with 100 μg/ml of peptide.

After peptide pulsing and washing 721.221 cells, they were co-incubated with SKW3-Vα3S1/Vβ13S1 T cells for 2 h with subsequent staining for upregulation of CD69 and flow cytometry (Fig. 3*A*). A comparative plot of the mean fluorescence intensity of the highest 100 μg/ml antigen concentration (Fig. 3*B*) demonstrated robust activation for the WT ADAMSTL5 epitope. The R5A and R8A mutations resulted in CD69 activation levels indistinguishable from negative control, while P4A still showed a slight degree of upregulation (Fig. 3*B*). Compared to the WT ADAMSTL5 peptide, the highly charged C2CD mimotope displayed the next highest activator potential (Fig. 3*B*).

Loss of any of the peptide arginine residues contributing to the electrostatic cluster reduced the activation potential of Vα3S1/Vβ13S1–expressing T cells.

### pHLA:TCR interface comparisons

**Figure 4:**
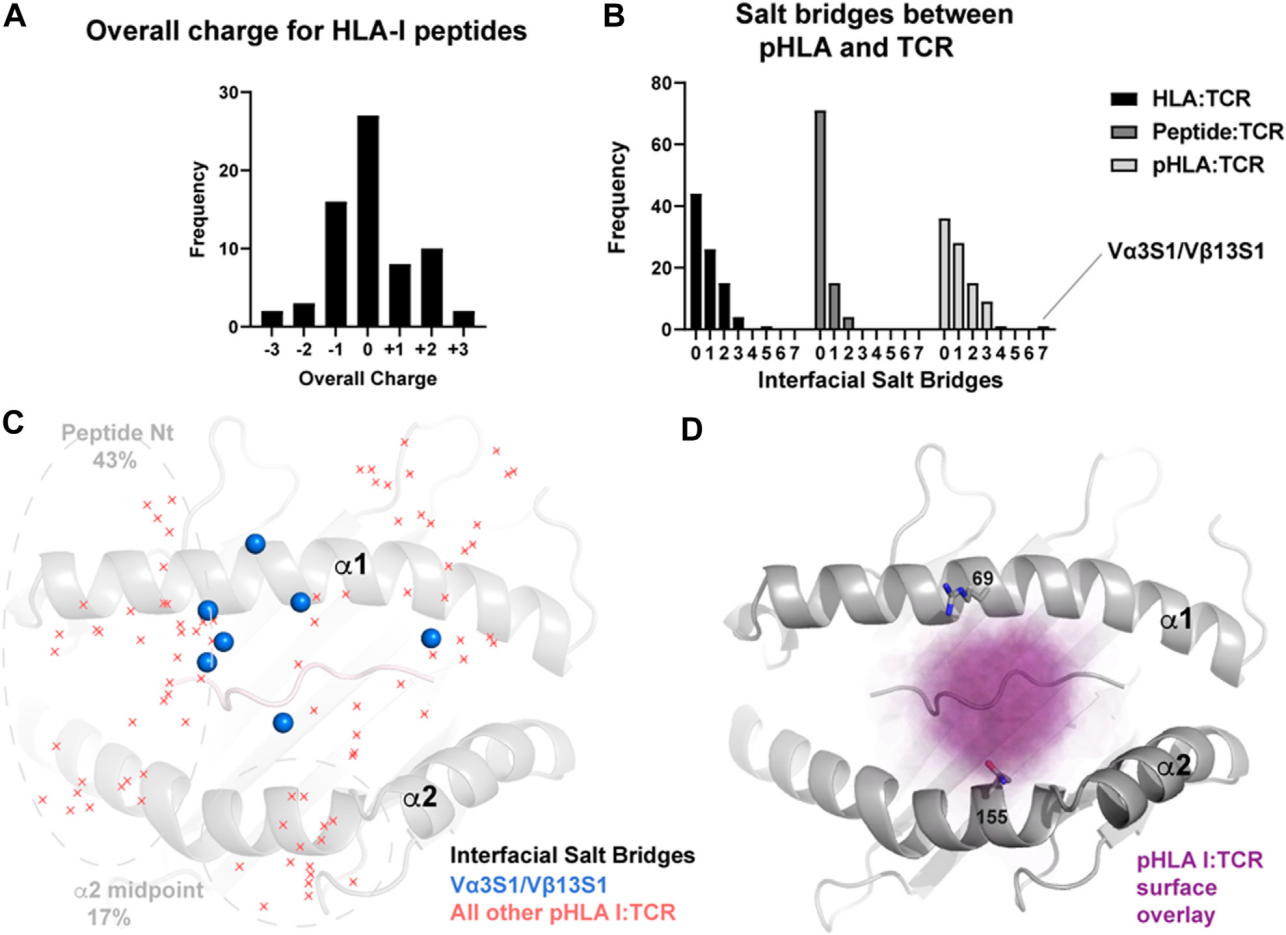
**Charge comparison with other known TCR-binding modes.***A*, frequency distribution of the total physiological charge for all currently characterized epitopes within TCR:MHC-I interactions. *B*, frequency distribution of pHLA:TCR interfacial salt bridges currently within the protein data bank. *C*, topological map of interfacial salt bridges for all aligned characterized class I pHLA:TCRs; *red crosses* indicate the position of the TCR charge and *blue points* indicate the same for Vα3S1/Vβ13S1. *D*, overlay of interfacial contacts, colored *purple* based on an alignment of all class I pHLA:TCRs. HLA, human leukocyte antigen; MHC, major histocompatibility complex.

The ADAMSTL5 epitope has four positively charged residues; thus, we next assessed the charges present in all previously characterized pHLA:TCR structures. Epitopes from all currently solved pHLA-I:TCR complexes were seen to be predominantly neutral or singly charged (Fig. 4*A*). The ADAMSTL5 epitope was observed as an outlier with its presentation of three positive charges towards the TCR interface.

Next, we sought to define to what extent the cross-interface salt bridges between Vα3S1/Vβ13S1 and HLA-C∗06:02 were unique for a TCR recognition system. We enumerated all such interactions in publicly available pHLA-I:TCR structures (Fig. 4*B*) excluding those with reversed polarity docking modes. The average number of ionic cross-interface interactions was 1.0 (with an SD of 1.04, Fig. 4*B*). With its total of seven cross-interface salt bridges, this marked the Vα3S1/Vβ13S1 recognition mechanism as the most charge-dominated to date.

We next sought to assess whether interfacial salt bridges are more likely to contribute toward binding particular regions of the HLA platform by enumerating the positions of all prior pHLA-I:TCR salt bridge interactions. Ionic contacts cluster at two points: near the epitope’s N-terminus (Fig. 4*C*) and the kink in the HLA platforms’ α2 helix. In contrast, Vα3S1/Vβ13S1’s ionic contacts appear more centrally directed nearer the peptide-binding groove.

We further overlayed the surface footprints for all class I pHLA:TCRs (Fig. 4*D*). The interaction density for the MHC platform is highest near the midpoint of the α1 helices between HLA residues 69 and 155. For 9-mer peptides which dominate the HLA-C∗06:02 repertoire, such interactions interrogate P3-P6. For the ADAMTSL5 peptide, the P8-Arg side chain also extends backwards into this target zone.

In conclusion, most currently characterized TCRs bind centrally near the epitope’s midpoint; to date, few charge-mediated TCR interactions have been observed within this zone.

## Discussion

The majority of previously characterized pHLA TCR docking modalities derive from HLA-A– or HLA-B–presented epitopes (29). Thus, while a diverse range of docking modes have been defined for other HLA allotypes (29), the only other comparators for this psoriatic TCR remains those specific for HLA-C∗08 (30, 31). Polar or hydrophobic interactions normally dominate the central portion of a TCR engagement interface in HLA-A and HLA-B with occasional charged interactions nearer the periphery. While the other characterized TCR–HLA-C∗08 complexes have higher than normal preponderance of charge-mediated contacts (30, 31), they are not to the degree observed here. We note that our work represents one of the few HLA-C–specific docking mechanisms; thus while the preponderance of salt bridge interactions used by Vα3S1/Vβ13S1 to engage HLA-C∗06:02 is notable, further work will be required to discern whether charge-dominated interfaces are more of a defining characteristic for psoriatic TCR interactions and/or HLA-C–specific ones.

The limited series of HLA-C–specific TCR complexes have thus far engaged the α1 helix extensively, forming anchoring contacts to its ^C∗^Arg-69. In HLA-A and HLA-B, residue 69 is a smaller Ala or Thr more often engaged in van der Waals contacts. In Vα3S1/Vβ13S1, a novel tetra-acidic “CDR-cage” actively embraces the ^C∗^Arg-69 charge, forming a network of seven salt bridges occurring across the pHLA-TCR interface. This extensive series of ionic interactions is uncommon among TCR-HLA structures (seven times the average) and is likely to cooperatively cluster following pHLA binding. This is potentially important as the entropic cost of dehydration for isolated cross-interface salt bridges may abrogate their contribution to binding affinity (32).

The outward-facing residues of the middle turns of each HLA-C α-helix include four positively charged residues (62, 66, 69, and 151). The presence of single arginine residues upon TCR-facing HLA surfaces is not uncommon (33); in HLA-A∗02–specific interactions, ^A∗^Arg-65 is a common participant. However, in this case, it is the only such participant and nearby Ala/Gly residues allow its side chain to compact against the helix such that its charge is more often directed towards solvent. However, in HLA-C, the coclustering of several large residues ^C∗^Arg-62, ^C∗^Gln-59, and ^C∗^Arg-69 seems to promote their upward projection such that they can instead probe the heterodimeric TCR region. This subtle difference may result in a higher preponderance of charge-dominated interactions for HLA-C–specific TCRs.

Is ADAMSTL5 likely a unique antigen for Vα3S1/Vβ13S1? In theory, peptide-HLA presentation of a 9-mer peptide allows ∼10^10^ potential permutations, amply sufficient to allow TCRs to specifically expand and identify pathological states. In practice, TCR cross-reactivity arises due to reuse of key protein sequences, the potential for different amino acids to be substituted in a given interaction, and obscuration of epitope positions during HLA display. Vα3S1/Vβ13S1 largely engages only the arginines within this peptide; it could potentially have reactivity against peptides with a similar level of charge.

Given this context, it is noteworthy that arginine citrullination homeostasis may be disturbed in hyperproliferative psoriatic lesions (34). The deimination of arginine concomitantly prevents its participation in charge-dominated interactions. Ishida-Yamamoto *et al.* (34) report that the activity of peptidylarginine deiminases is suppressed within hyperproliferative psoriatic epidermis increasing the arginine content of skin proteins such as Keratin K1. Reversion to genomically templated charge-rich sequences through disturbance of normal posttranscriptional modification patterns may thus be a theoretical mechanism by which self-antigens could arise.

We conclude that HLA-C∗06:02 displays an unusually positively charged antigen presentation interface, and its cleft is ideally suited for presentation of epitopes from the extracellular matrix protein ADAMSTL5. The combined electropositivity of peptide and HLA is exquisitely matched by opposite charges within an isolated psoriatic TCR providing the most charged pHLA:TCR interface to date. Arginine side chains are thrust towards potential TCRs by both HLA-C∗06:02 and the psoriatic antigen ADAMSTL5; these key features may help further identify defining features of psoriatic TCRs and, following further verification, may potentially hold therapeutic significance.

## Experimental procedures

### Protein expression and purification

The extracellular portion of HLA-C∗06:02, human β-2-microglobulin (β2m), and the α-chain and β-chain of Vα3S1/Vβ13S1 were cloned into pET30 and expressed into inclusion bodies within *Escherichia coli.* Inclusion bodies were harvested and solubilized in 20 mM Tris–HCL pH 8.0, 6 M guanidine hydrochloride, 1 mM EDTA, 1 mM DTT, and 0.2 mM PMSF. HLA-C∗06:02 heavy chain was refolded in the presence of human β2m (120 mg and 30 mg, respectively) and 10 mg of the self-peptides (VRSRR-*C*-LRL) where an aminobutyric acid residue (*C*) is substituted in place of cysteine in the P6 position (22). Vα3S1/Vβ13S1 was refolded separately at 1:1 ratio of individual TCR chains.

Refolds were carried out in 1 L refold buffer (0.1 M Tris–HCL pH 8.0, 2 mM EDTA, 0.4 M L-arginine, 0.5 mM oxidized glutathione, 5 mM reduced glutathione, and 0.2 mM PMSF). The refold solution was then dialyzed into 20 mM Tris–HCL pH 8.0. Refolded protein was initially purified using DEAE-sepharose anion exchange followed by size-exclusion chromatography in 20 mM Tris pH 8.0, 150 mM NaCl. The final purification step was anion-exchange chromatography with a HiTrap Q column with a linear gradient of 20 mM Tris pH 8.0, 0 to 1 M NaCl. Samples were buffer exchanged to 20 mM Tris pH 8.0, 150 mM NaCl for storage prior to use.

Vα3S1/Vβ13S1 TCR was cocomplexed with a stoichiometric amount of HLA-C∗06:02-VRSRR***C***LRL and incubated for 30 min at room temperature prior to purification by gel-filtration chromatography.

### Crystallization and X-ray data collection

Crystals were obtained by hanging drop experiments with Vα3S1/Vβ13S1:HLA-C∗06:02-VRSRR***C***LRL complex at 12 mg/ml incubated over a reservoir containing 20% w/v PEG 3350, 0.2 M NaSO_4_. Prior to flash freezing in liquid nitrogen, crystals were cryo-protected in reservoir solution supplemented with 20% v/v ethylene glycol. All X-ray diffraction data were obtained at the Australian synchrotron MX2 beamline.

### Structure determination and refinement

Diffraction data were processed with XDS (35) and then scaled and merged with AIMLESS (36). Phases were obtained by molecular replacement using PHASER (37) with HLA-C∗06:02 used as the initial search model (Protein data bank [PDB]: 5W67 (20)) followed by rounds of restrained refinement in PHENIX (38) and model building with COOT (39). A single round of simulated annealing was performed to minimize bias, and TLS was applied to final rounds of refinement. Data collection and refinement statistics for all structures are summarized in Table 1 and deposited with www.rcsb.org under PDB code 8SHI.

### Structural pHLA:TCR comparisons

Examples of class I pHLA:TCR structures filtered for redundancy were downloaded from the protein data bank and aligned based on the platform of the MHC heavy chain. Frequency distributions were determined after defining an interfacial salt bridge as being a sub 4 Å approach between two oppositely charged residues. Surface contacts were defined as within 4.2 Å, each surface overlay represented 1.5% opacity. PDBs used for the analysis are as follows: this study and 7n6e, 7n1f, 7n1e, 6vrn, 6vrm, 6vqo, 6tro, 6rsy, 6rpb, 6rpa, 6rp9, 6amu, 6am5, 5yxn, 5tez, 5nmg, 5nme, 5nht, 5men, 5jhd, 5isz, 5eu6, 5e9d, 5e6i, 5d2n, 5d2l, 5c09, 5c08, 5c07, 5c0c, 5c0b, 5c0a, 5bs0, 5brz, 3utt, 3qdm, 3qdg, 3pwp, 3o4l, 3hg1, 3h9s, 3gsn, 2bnr, 1oga, 1bd2, 1ao7, 4eup, 5wkh, 3vxm, 3vxs, 5avf, 6avg, 6vmx, 1mi5, 3ffc, 3sjv, 4qrp, 4g9f, 3mv7, 6bj2, 4jrx, 6mtm, 3dxa, 3kpr, 3kps, 4mji, 2ypi, 6uon, 3pqy, 5ivx, 5m02, 5wlg, 6g9q, 7jwj, 1fo0, 1g6r, 1kj2, 1mwa, 2ol3, 2oi9, 3tf7, 3tfk, 3tpu, 4mvb, 4mxq, 4n0c, 4n5e, 6191, 6jtn, 6uli.

### Surface plasmon resonance affinity measurements

A BIAcore T200 was used for surface plasmon resonance affinity measurement. Two independent experiments using different batches of protein were performed at 298 K. The buffer used contained 20 mM Tris pH 8.0, 300 mM NaCl, and 0.005% surfactant P20 (TBS-300-P20). The HLA-C∗06:02 or alanine mutant was amine-coupled to flow cells of a CM5 sensorchip (BIAcore). Vα3S1/Vβ13S1 TCR was serially diluted in TBS-300-P20 and passed simultaneously over the test (HLA-C∗06:02), and empty flow cells surfaces at a flow rate of 5 μl/min. All measurements were taken in duplicate. All obtained data were analyzed using Prism (GraphPad) using steady-state equilibrium analysis.

### CD69 upregulation assays

Activation of SKW3.Vα3S1/Vβ13S1 cells (1 × 10^5^) was assessed using cell-surface CD69 upregulation after 17 to 20 h incubation with either 721.221 parental or 721.221-HLAC∗06:02 (1:1 ratio) cells preincubated for 1 hour with different peptides. Following co-incubation, the SKW.TCR cells were co-incubated for 17-20 hours with antigen presenting cells following 1 hour preincubation with peptide. For all experiments, SKW3.TCR cells alone served as negative control. Flow cytometry data were acquired and analyzed as described previously (40). The CD69 expression profiles were measured as geometric mean fluorescence intensity. A maximum of 50,000 lymphocytes were acquired on a BD LSRII flow cytometer utilizing BD FACSDIVA software (FlowCore, Monash University) and analyzed using FlowJo software (version 10, BD, www.flowjo.com/).

## Data availability

Atomic coordinate and structure factors of the Vα3S1/Vβ13S1: HLA-C∗06:02-VRSRR***C***LRL ternary complex were deposited in the Protein Data Bank (PDB) under the ID 8SHI.

## Supporting information

This article contains supporting information (30).

## Conflict of interest

D. G. B is an employee of Janssen Pty Ltd. All other authors declare that they no conflicts of interest with the contents of this article.
