# Supplementary material for: Complimentary electrostatics dominate T-cell receptor binding to a psoriasis-associated peptide antigen presented by human leukocyte antigen C∗06:02
Source: J Biol Chem. 2023 Jun 15;299(7):104930. doi: 10.1016/j.jbc.2023.104930 (PMC10371836; doi:10.1016/j.jbc.2023.104930)
Supplement: Supporting Figures S1 and S2 and Tables S1–S3 [file mmc1.pdf]

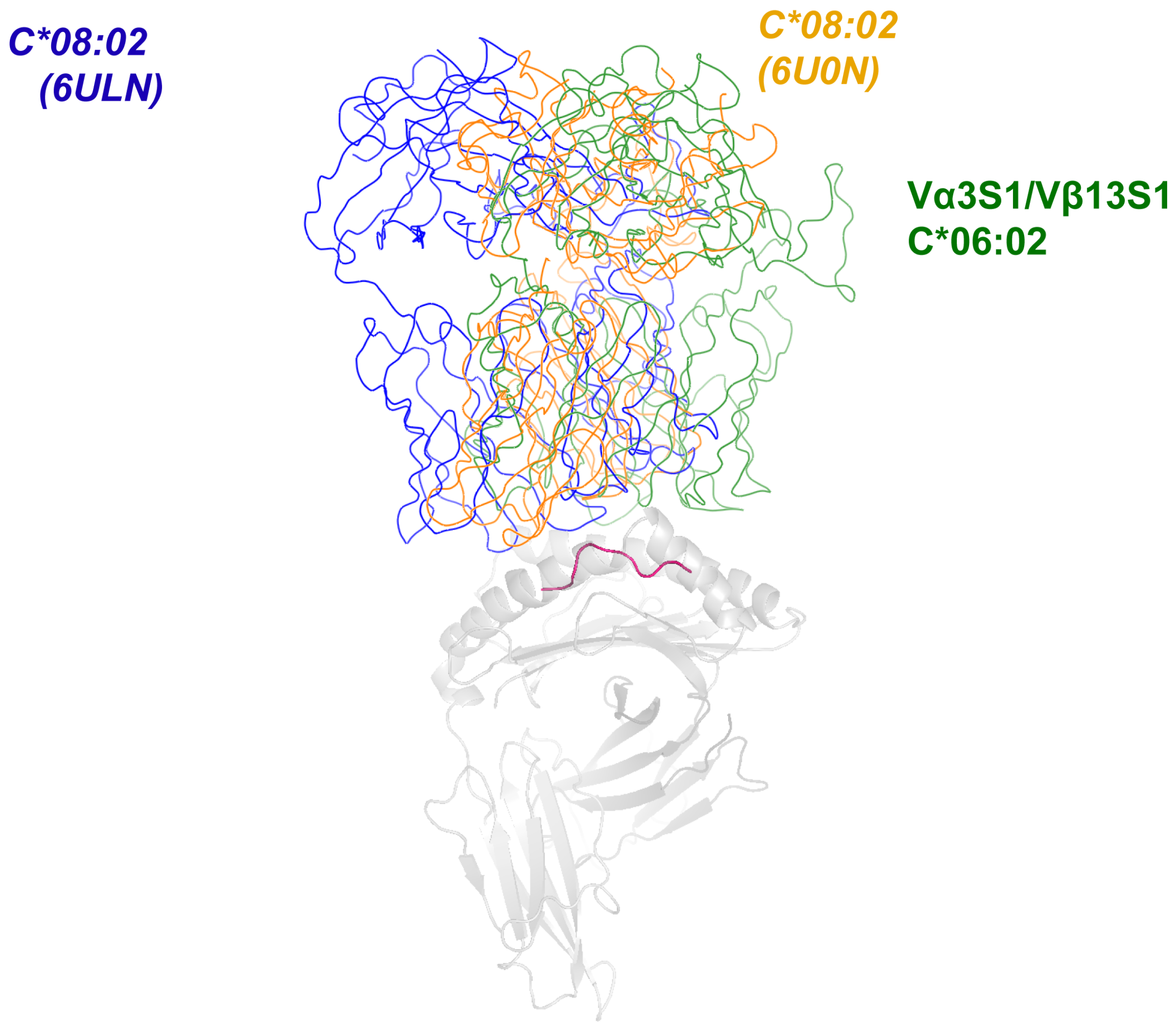

Supplementary Figure 1 – HLA-C TCR docking comparision
Overlay of different docking modes currently identified for HLA-C restricted TCRs after aligning the pHLA. The TCRs are displayed with ribbon representation and the HLA as cartoon. TCRs shown are *green* Vα3S1/Vβ13S1 in complex with HLA-C*06:02 (this work), *orange* TCR10:HLA-C*08:02(GADGVGKSAL) and *blue* TCR9d:HLA-C*08:02(GADGVGKSAL). The latter two structures are from Sim *et al.* 2020.

**Peptide**	**TCR contact**	**Interaction type**
P4-Arg Gdn	$^{TCR\alpha}$Arg-59 Gdn	π-π
P4-Arg C_{β}	$^{TCR\beta}$Glu-99 C_{γ}	vdW
P5-Arg Gdn	$^{TCR\beta}$Tyr-95 Ring	π-π
P5-Arg $N_{\eta1}$	$^{TCR\beta}$Asp-100 $O_{\delta1}$, $O_{\delta2}$	Salt-bridge
P8-Arg O	$^{TCR\beta}$Tyr-95 OH	vdW
P8-Arg C_{β}	$^{TCR\beta}$Tyr-95 OH	vdW
P8-Arg C_{γ} / C_{δ}	$^{TCR\beta}$Tyr-95 Ring	vdW
P8-Arg N_{ε} / C_{ζ}	$^{TCR\beta}$Tyr-95 O	vdW
P8-Arg $N_{\eta1}$	$^{TCR\beta}$Ser-96 Cα / C_{β}	vdW
P8-Arg $N_{\eta2}$	$^{TCR\beta}$Glu-97 $O_{\varepsilon1}$	Salt-bridge
**HLA C*06:02 α1 helix**	**TCR contact**	**Interaction type**
^{C*06}Lys-80 N_{ζ}	$^{TCR\beta}$Glu-30 $O_{\varepsilon1}$ / $O_{\varepsilon2}$	Salt-bridge
^{C*06}Arg-79 NH1	$^{TCR\beta}$Ala-42 O	H-bond
^{C*06}Arg-79 NH2	$^{TCR\beta}$Ala-42 C_{β}	vdW
^{C*06}Glu-152 $O_{\varepsilon1}$	$^{TCR\beta}$Tyr-95 OH	H-bond
^{C*06}Arg-69 C_{γ} / C_{δ}	$^{TCR\beta}$Glu-97 $O_{\varepsilon1}$ / $O_{\varepsilon2}$	vdW
^{C*06}Arg-69 C_{ζ}	$^{TCR\beta}$Glu-97 O	vdW
^{C*06}Arg-69 NH1	$^{TCR\beta}$Glu-99 $O_{\varepsilon1}$	Salt-bridge
^{C*06}Arg-69 NH1	$^{TCR\alpha}$Asp-101 $O_{\delta1}$, $O_{\delta2}$	Salt-bridge
^{C*06}Arg-69 NH2	$^{TCR\alpha}$Asp-101 $O_{\delta1}$	Salt-bridge
^{C*06}Gln-65 $O_{\varepsilon1}$	$^{TCR\alpha}$Asn-39 $N_{\delta2}$	H-bond
^{C*06}Gln-65 $N_{\varepsilon2}$	$^{TCR\alpha}$Ala-102 O	H-bond
^{C*06}Gln-65 $N_{\varepsilon2}$	$^{TCR\alpha}$Tyr-104 O	H-bond
^{C*06}Gln-62 NH1 /2	$^{TCR\beta}$Glu-99 $O_{\varepsilon1}$ / $O_{\varepsilon2}$	Salt-bridge
^{C*06}Gln-62 N_{ε} / C_{ζ} / C_{δ}	$^{TCR\alpha}$Asn-39 C_{β} / $C_{\gamma / N\delta2}$	vdW
^{C*06}Gln-62 N / Cα	$^{TCR\alpha}$Leu-103 $C_{\delta2}$	vdW
^{C*06}Gln-61 O	$^{TCR\alpha}$Ser-105 C_{β}	vdW
^{C*06}Gln-61 $O_{\delta1}$	$^{TCR\alpha}$Ser-105 O_{γ}	H-bond
**HLA C*06:02 α2 helix**	**TCR contact**	**Interaction type**
^{C*06}Glu-153 $O\varepsilon_{1}$	$^{TCR\beta}$Tyr-95 OH	H-bond
^{C*06}Trp-147 $N\varepsilon_{1}$	$^{TCR\beta}$Tyr-95 OH	wdW
^{C*06}Trp-147 $C_{\delta1}$	$^{TCR\beta}$Tyr-95 OH	wdW
^{C*06}Lys-146 C_{γ} C_{δ}, Cε	$^{TCR\beta}$Asn-28 C_{γ}, $N_{\delta2}$	wdW

Interactions classified as < 4 Å, vdW = van der Waals

Supp. Table 1 TCR pHLA interactions

Table 2: SPR analysis on HLA-C6*02:01 mutations on α1 helix: n=2

α1 helix			
Replicates	WT	R62	Effect on mutation
n=1	2.881 ± 0.48	28.26 ± 1.8	**
n=2	9.053 ± 0.28	16.68 ± 4	*
	WT	Q65	
n=1	13.8 ± 0.59	> 150	***
n=2	9.775 ± 0.43	>375	***
	WT	R69	
n=1	11.78 ± 0.92	> 779	***
n=2	43.45 ± 4.4	> 879	***
	WT	Q72	
n=1	11.78 ± 0.92	16.74 ± 0.77	*
n=2	9.053 ± 0.28	9.762 ± 1	*
	WT	V76	
n=1	10.78 ± 0.32	>1894	***
n=2	7.104 ± 2	>1479	***
	WT	R79	
n=1	19.05 ± 1.4	9.76 ± 0.77	*
n=2	11.55 ± 0.39	5.293 ± 1.2	*
	WT	K80	
n=1	19.05 ± 1.4	56.32 ± 2.8	*
n=2	9.775 ± 0.43	25.03 ± 1.6	*

Table 3: SPR analysis on HLA-C6*02:01 mutations on α2 helix: n=2

α2 helix			
Replicates	WT	R169	Effect on mutation
n=1	10.78 ± 0.32	12 ± 0.5	*
n=2	13.21 ± 0.59	7.124 ± 1.2	*
	WT	T163	
n=1	9.053 ± 0.28	6.78 ± 0.88	*
n=2	9.053 ± 0.28	6.376 ± 1.3	*
	WT	Q155	
n=1	9.775 ± 0.43	6.559 ± 2	*
n=2	9.775 ± 0.43	3.635 ± 2.1	*
	WT	R151	
n=1	10.78 ± 0.32	4.487 ± 0.99	*
n=2	13.21 ± 0.59	8.861 ± 1.1	*
	WT	K146	
n=1	9.053 ± 0.28	>200	***
n=2	15.14 ± 0.57	>3756	***
	WT	R145	
n=1	9.775 ± 0.43	3.509 ± 1.4	*
n=2	11.55 ± 0.39	12.04 ± 1.9	*
	WT	I142	
n=1	43.45 ± 4.4	10.24 ± 5	**
n=2	7.104 ± 2	5.316 ± 2.7	*

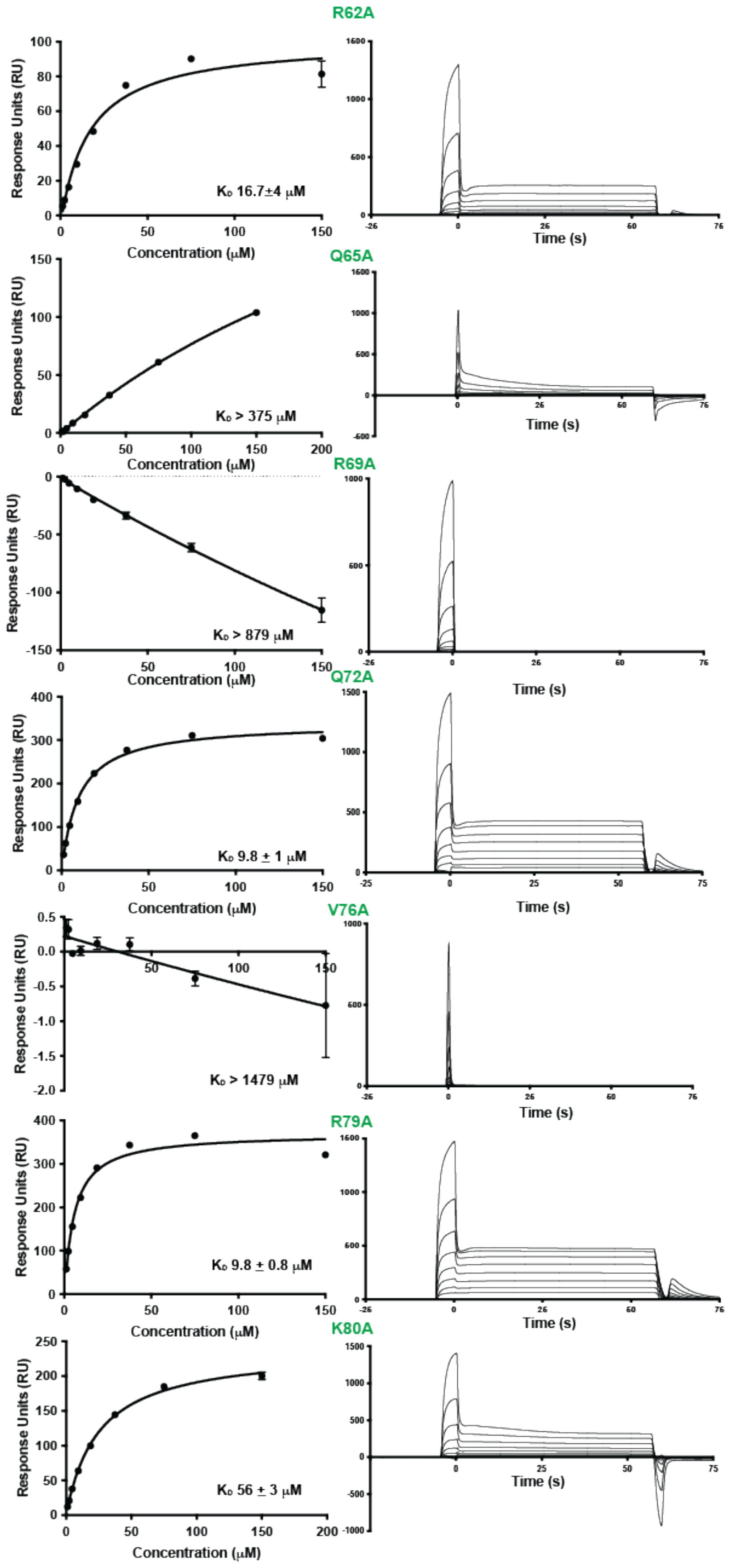

Supplementary Figure 2a – Alanine-scanning mutagenesis of the HLA C6*02 helix 1

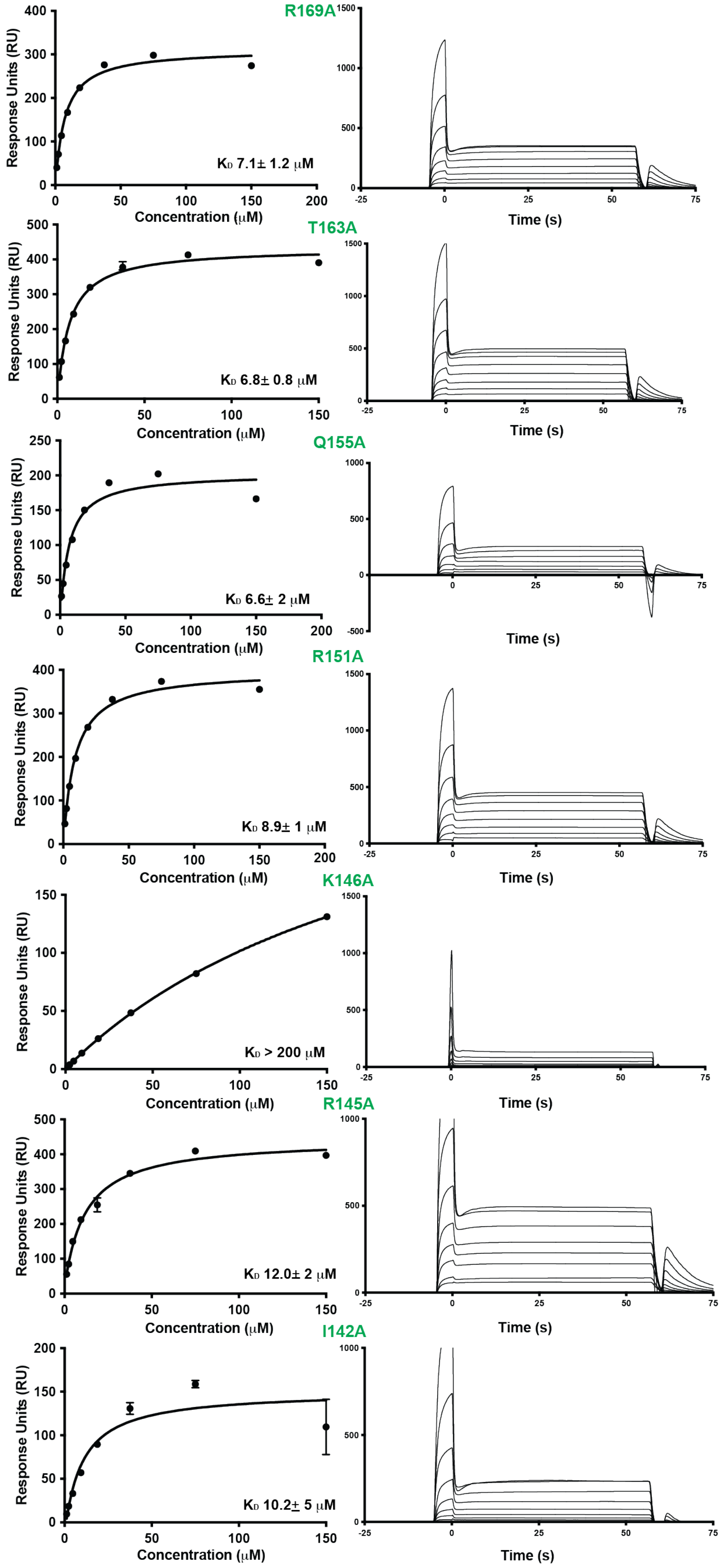

Supplementary Figure 2b – Alanine-scanning mutagenesis of the HLA C6*02 helix 2
